# Biomimetics—Prospects and Developments

**DOI:** 10.3390/biomimetics7010029

**Published:** 2022-02-25

**Authors:** Ille C. Gebeshuber

**Affiliations:** Institute of Applied Physics, Vienna University of Technology, Wiedner Hauptstrasse 8-10/134, 1040 Wien, Austria; gebeshuber@iap.tuwien.ac.at; Tel.: +43-1-58801-13483; Fax: +43-1-58801-13499

Since its inaugural issue published in 2016, the scientific journal *Biomimetics* (ISSN 2313-7673), published by MDPI, has developed into a robust scientific journal that is appreciated in the field. *Biomimetics* is an open access journal regarding biomimetics, bioinspiration and bionics, dedicated to research that relates to the most basic aspects of living organisms and the transfer of their properties to human applications. The journal aims to provide a forum and survey for researchers and professionals in the fields of materials science, mechanical engineering, nanotechnology and biomedicine interested in exploiting biologically inspired designs in engineering systems, as well as technology and biomedicine aimed to developing novel solutions that enable sustainable innovation. *Biomimetics* invites submissions on a wide range of topics, including, but not limited to: biomimetic mechanism and design, biomimetic robotics, biofabrication and characterization, biomimetic and bioinspired chemistry, biosensing, nanotribology, nanomechanics, micro/nanoscale studies, plant biomechanics, synthetic systems, self-organization and cooperative behavior, tissue engineering and bioinspired materials.

The success of *Biomimetics* is based on publishing articles written by specialist scientists as well as experts who work in an inter- or transdisciplinary way in all fields of biomimetics, including professional education, research and development. The journal has contributed immensely to consolidating biomimetics as a field of research and supports it in its growth. I wish to convey my warmest congratulations to the journal *Biomimetics* on the occasion of receiving its first impact factor in 2022, and keenly look forward to witnessing the continuous achievements of the journal along its mission of being dedicated to the advancement of communication and cooperation among all scholars, and the dissemination of knowledge and education in the field of biomimetics.

Biomimetics is a field that is a little bit different from various other scientific fields, because of its inherent inter- and transdisciplinarity. Biomimetics experts understand the basic biology and current challenges. Furthermore, their goal is to abstract and transfer biological principles into engineering, architecture or art—and eventually in a way the resulting products and processes are sustainable. Such a broad and at the same time deep approach is difficult to realize in a single person. Biomimetics as a field is very attractive to students and the general population. The challenge is how to teach and communicate biomimetics in a way that creates a positive effect for science and society, on various levels. 

Living nature provides us a glimpse into the possible, into what can be done. Additionally, with curiosity, eagerness and dedication, biomimetics experts can learn from this great master [1]. Conventional innovation generally is incremental—it aims to improve technology by making it faster, cheaper or smaller/larger (i.e., maximization of one or few parameters) instead of optimizing the whole. On the other hand, innovation based on biomimetics can progress by making technology or architecture or the arts better, via respect and love for the biosphere—if sustainability aspects are considered on a deep level. We all, the editors, authors and readers, are the ones to shape the journal for the future, and contribute to determine the way we are going to approach this amazing field.

When a journal receives its first impact factor, it is a time for reflecting on the past and looking ahead to the future. For the last six years, *Biomimetics* has made valuable contributions to serve as a platform for biomimetics experts around the world—for informing their colleagues and being informed. It has further contributed to combining science with values, resulting in approaches that focus on a sustainable future [2,3], a good life for all, the importance to care for the biosphere, innovation [2], and how to learn from a great master via a look into the possible, into what can be done, with curiosity, dedication, devotion, eagerness, interest, engagement and respect.

Organisms can be viewed from various angles. When looking at organisms and ecosystems as a biomimetics expert, one realizes the combination of beauty and expediency. Viewed from a purely rational viewpoint, nature’s “technology” can be seen as so far advanced that it allows us a look into the possible, into what can be done, combined with best practice examples that actually work. Some of these almost perfect solutions might even exceed our intellectual horizon, and we might never be able to grasp them completely. In this way, by doing biomimetics, our minds stay challenged. Biomimetics allows us to enter completely new fields, and to widen our horizon, and potentially even to cross current physical and epistemological boundaries.

Very important in this approach is to ask the right questions! To see what puzzles you. “Why do birds migrate at night?” “Why are there black butterflies in the hot tropics?” “How can we improve stretch foils used in packaging?” and develop research questions therefrom. The new, the unexpected, the disruptive solutions often originate from observations that seem strange and interesting.

For such an approach, we need good broad education [4], because biomimetics experts need to be all-round experts with significant general knowledge, and potentially have an idea how to translate the research into something that can be used.

Biomimetics research opens minds for interdisciplinary approaches, unusual thinking, new ways of doing things, and may result in disruptive new products and processes. Especially in times of biological transformation of the manufacturing industries for biointelligent value creation [5], the biomimetic approach is of ultimate importance and will be further refined and optimized in the years to come. 

For the remainder of this article, three examples shall illustrate the prospects and developments of modern biomimetics research at different technology readiness levels (TRL): firstly, the exquisite sense of magnetoreception in birds (which is potentially based on quantum physics) is exemplified (low TRL 1-2, basic technological research), secondly, disruptive cooling technologies (TRL 2-3, research to prove feasibility), inspired by passive radiative cooling as seen in desert ants and further organisms, are introduced, and thirdly, the industrially highly relevant research (medium TRL 4, technological development) concerning bioinspiration related to the development of efficient, biobased and recyclable stretch foils for commercial applications is summarized. For further examples, see the article “A gaze into the crystal ball: Biomimetics in the year 2059” [6].

The European robin *Erithacus rubecula* (Figure 1) is a little bird that is known to most who live in Europe. It starts to sing about one hour before dawn and sings throughout the day. European robins who live in Northern and Eastern Europe migrate in winter to the Mediterranean or to the Middle East. The European robin has played an important role in the discovery and scientific recognition of the magnetic sense [7,8,9]. As Xu and co-workers report, the proportions of the quantum mechanical singlet and triplet states of radical pairs in the photoreceptor cells in the bird’s retina are sensitive to the Earth’s magnetic field and might be the clue to the sensitive magnetoreception in the migratory European robin. Quantum physics might also be necessary for proper functioning of photosynthesis, olfaction and enzymatic activity [10] as well as of eyes [11], technical organic solar cells [12] and DNA mutations [13]. 

Especially from organisms who live in extreme environments, one can learn for our normal world, by pushing boundaries and by turning towards counterintuitive approaches. One example is the development of the PCR chain reaction by Nobel prize winner Karry Mullis, which was inspired by thermophilic bacteria, extremophiles that thrive at temperatures well above the boiling point of water [14]. The PCR tests with their great sensitivity that resulted from that research are of utmost importance in the Corona pandemic.

Not exactly thermophilic, but foraging during noon in the hot and dry Sahara in Northern Africa, are the Saharan desert ants *Cataglyphis* spp. They are the fastest ants on Earth, and can sustain a body temperature well above 50 °C (122 °F), with surface temperatures of up to 70 °C (158 °F). The Sahara silver ant *Cataglyphis bombycina* has silver hairs (Figure 2) with photonic structures on the top and sides of its body that reflect light (visible and infrared) and that also help emit body heat in the mid-infrared spectral band by passive radiative cooling [15], by offloading excess heat via thermal radiation being emitted from the hot ant bodies to the cold sky [16]. Various publications deal with biomimetic technology inspired by the cooling properties of the desert ant. Wu and co-workers [17], for example, fabricated photonic structures on polydimethylsiloxane, similar to the silver hairs of the desert ants. Considerably enhanced optical reflection was observed as well as slightly improved mid-infrared emission. The temperature of glass bottles used in the experiment was reduced by around 5.6 °C in the hot daytime and then kept relatively warm in the cold nighttime. Exciting technical developments regarding biomimetic passive radiative cooling of façades, space equipment and electronic equipment can be envisaged. 

Recent papers on cooling in *Biomimetics* deal with an assessment of zebra-stripes-based strategies in the energy performance of buildings [18], biomimetic strategies for urban heat island mitigation [19], evaporative cooling related to façades [20], biomimetics to address climate-related energy building challenges in Panama [21], a biomimetic groundwork for thermal exchange structures inspired by plant leaf design [22], biomimetics of the heat reduction effect seen in *Nepenthes alata* (a tropical pitcher plant from the Philippines) [23] and technical nanostructures inspired by the hydrophilic and superhydrophilic properties of certain plants [24].

The Research, Technology and Innovation Initiative “Production of the Future” of the Austrian Federal Ministry for Climate Action, Environment, Energy, Mobility, Innovation and Technology promotes the development of Austrian manufacturing research by focusing on cooperation between industry and science. Global plastics consumption is expected to double within the next 20 years. Around 25.8 million tons of plastic waste are produced annually in Europe, while the recycling rate is stagnating at around 30%. Packaging waste, including stretch wrap for pallet packaging, accounts for 59% of all plastic waste produced. Stretch film consumption is expected to increase even more in the future. One attempt to address this problem is the development of more efficient, bio-based and recyclable stretch foil [25] (Figure 3). The objective of such a stretch foil is to reduce the use of stretch wrap based on fossil raw materials for packaging and securing of load carriers by 30% until 2025 compared to 2016. Anticipated project results related to biomimetics are a demonstrator-like prototype of a bio-based, recyclable and biomimetically functionally structured stretch wrap, proof of function in a laboratory environment as well as a mathematical model to develop and optimize a functional and biomimetic structuring of the stretch wrap by means of simulation.

Packaging and compostability are also topics of interest for *Biomimetics*. A related recent paper on compostable materials describes additive manufacturing and bio-welding of mycelium-based composites. Such fungi-based materials are of growing interest for research and also the industry due to their light weight, compostable and regenerative features [26]. The design of environmentally friendly packaging materials that display greater resistance to degradation in the presence of moisture and UV light stimulated research that deals with the degradation behavior of nanocomposites consisting of poly(lactic acid) PLA and carbon nanotubes [27]. PLA is commonly known as corn plastic and is recyclable, biodegradable and compostable.

The remainder of this editorial highlights two aspects of biomimetics that are arguably fundamental to its future success in science and society: the first one is resource-related, and the second one information-related. On various levels, resource scarcity poses a major challenge to current industries, and the dependence of industrialized countries on scarce resources from countries where people and/or nature are not treated decently might diminish needed actions on the humanitarian, environmental and political level because of industrial dependences. Scarcity is a concept that is common in the industry world and in animated Nature. Organisms have learnt to deal with it over millions of years. They use local materials, and—opposed to technical systems, where a wide range of chemical elements from around the globe is currently used to obtain certain functions—related functions in organisms are often realized with less different chemical elements, but refined smart structuring of a limited number of base materials (Figure 4) [28]. One area where such a structure-based approach is already realized is in microelectromechanical systems (MEMS) development. In this field, only a handful of materials can be used, and the designer has to work with a structure-rather-than-material approach [29]. Currently, the elemental composition of technical devices is distinctively different from the one in organisms, resulting in resource scarcity and dependences. Moving towards a biological transformation of technology might disruptively change our use of materials in technical devices and processes towards that of organisms [5].

The second aspect of biomimetics that is arguably fundamental to its future success is related to the complexity of the physics utilized in organisms. Contrary to potential ordinary belief, the complexity of the physics of organisms does not correlate with their size or location in the phylogenetic tree. Single celled algae or bacteria can be as great an inspiration for new approaches in biomimetics as the largest whale or the most complex ecosystem. This allows knowledge gain in a vast variety of research environments and promises great future developments!

## Figures and Tables

**Figure 1:**
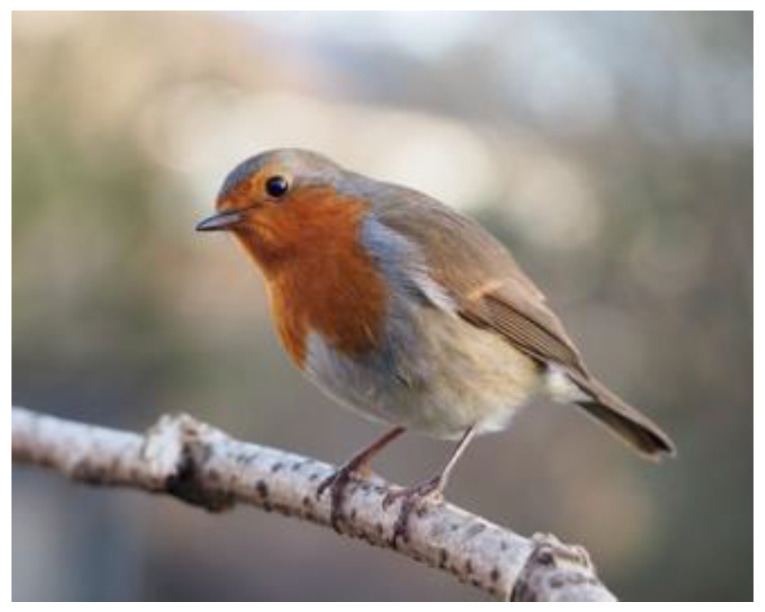
European robin *Erithacus rubecula*. Research on its magnetic sense exemplifies the importance of quantum physics in biology. By © Francis C. Franklin/CC-BY-SA-3.0, https://commons.wikimedia.org/w/index.php?curid=31367900 (accessed 27 January 2022).

**Figure 2:**
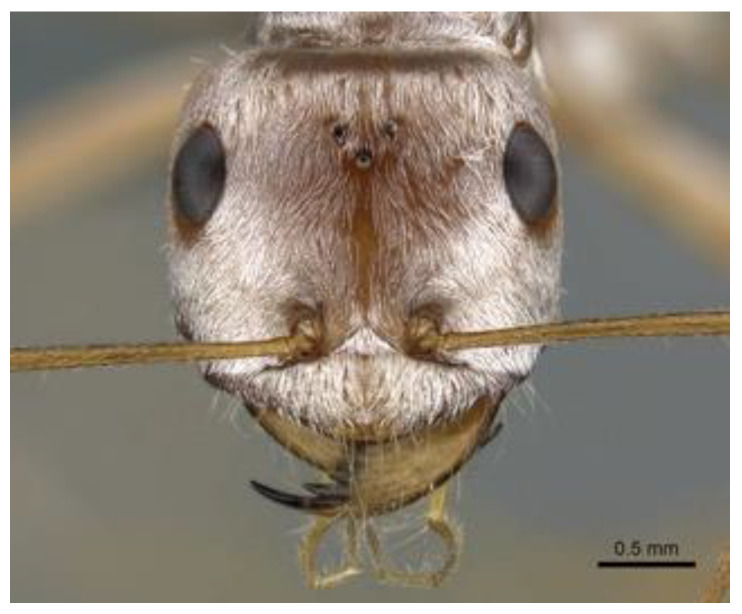
Saharan silver ant *Cataglyphis bombycina*. Research on its passive radiative cooling abilities inspires new approaches to cooling. Photo by Estella Ortega/URL: https://www.antweb.org/bigPicture.do?code=casent0906667&shot=h&number=1%C2%AEionName=Africa (accessed 27 January 2022). Image Copyright © AntWeb 2002–2022. Licensing: Creative Commons Attribution License.

**Figure 3:**
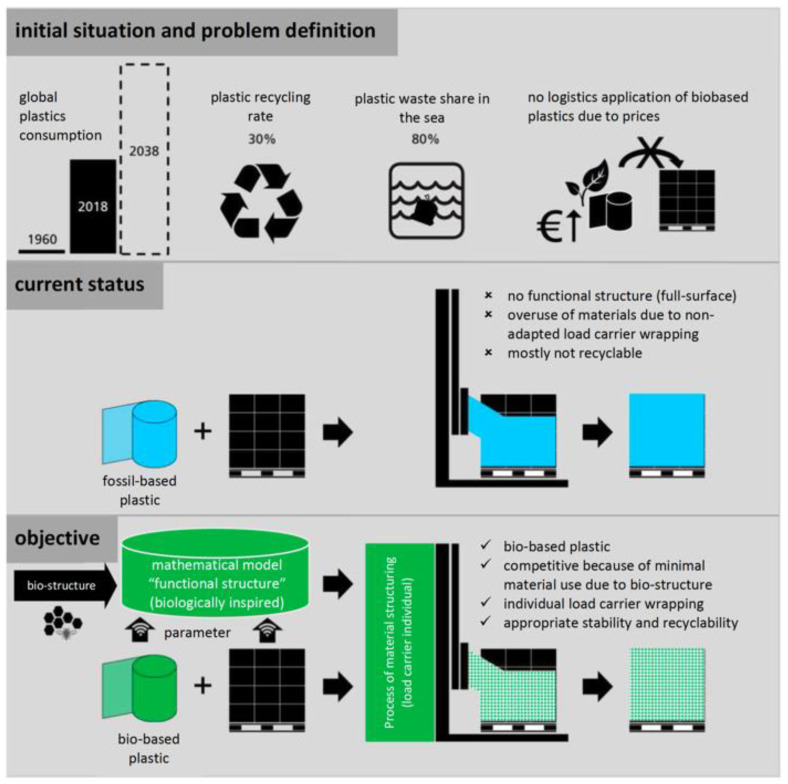
Biomimetics is paramount in the development of more efficient, bio-based and recyclable stretch foil that aims at a reduction of the use of stretch wrap based on fossil raw materials for packaging and securing of load carriers by 30% until 2025 compared to 2016. EFFIE_KickOff_20190418 © Fraunhofer Austria. Image reproduced with permission.

**Figure 4:**
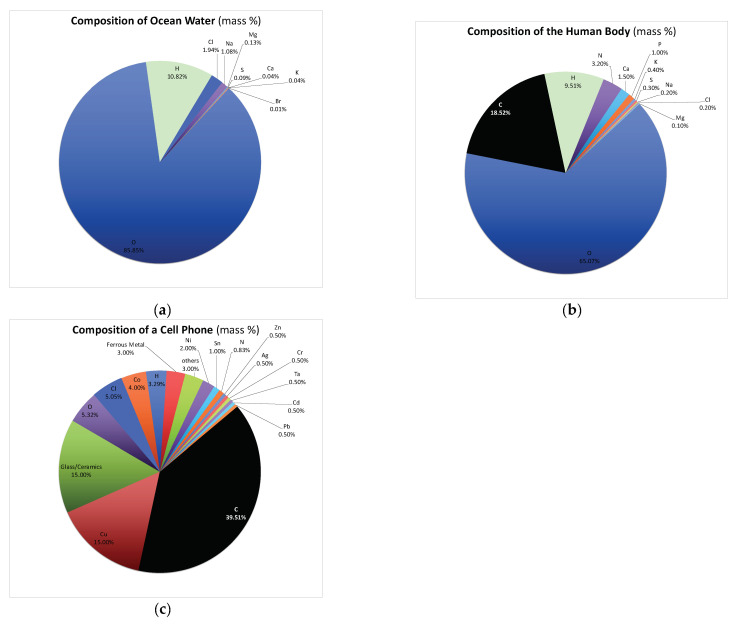
The elemental compositions of ocean water, the human body and a cell phone. The elemental composition of ocean water (**a**) is similar to the elemental composition of the human body (**b**) and very different from the composition of current engineering devices such as a cell phone (**c**). Novel biomimetic processes might in the future shift the elemental composition of technical devices towards these of organisms, reducing the need for scarce resources.
